# A Study on Differences between Simplified and Traditional Chinese Based on Complex Network Analysis of the Word Co-Occurrence Networks

**DOI:** 10.1155/2020/8863847

**Published:** 2020-12-03

**Authors:** Zhongqiang Jiang, Dongmei Zhao, Jiangbin Zheng, Yidong Chen

**Affiliations:** ^1^China Mobile (Suzhou) Software Technology Co., Ltd., Suzhou, China; ^2^Department of Artificial Intelligence, School of Informatics, Xiamen University, Xiamen 361005, China; ^3^Xiamen Key Laboratory of Language and Culture Computation, Xiamen University, Xiamen 361005, China

## Abstract

Currently, most work on comparing differences between simplified and traditional Chinese only focuses on the character or lexical level, without taking the global differences into consideration. In order to solve this problem, this paper proposes to use complex network analysis of word co-occurrence networks, which have been successfully applied to the language analysis research and can tackle global characters and explore the differences between simplified and traditional Chinese. Specially, we first constructed a word co-occurrence network for simplified and traditional Chinese using selected news corpora. Then, the complex network analysis methods were performed, including network statistics analysis, kernel lexicon comparison, and motif analysis, to gain a global understanding of these networks. After that, the networks were compared based on the properties obtained. Through comparison, we can obtain three interesting results: first, the co-occurrence networks of simplified Chinese and traditional Chinese are both small-world and scale-free networks. However, given the same corpus size, the co-occurrence networks of traditional Chinese tend to have more nodes, which may be due to a large number of one-to-many character/word mappings from simplified Chinese to traditional Chinese; second, since traditional Chinese retains more ancient Chinese words and uses fewer weak verbs, the traditional Chinese kernel lexicons have more entries than the simplified Chinese kernel lexicons; third, motif analysis shows that there is no difference between the simplified Chinese network and the corresponding traditional Chinese network, which means that simplified and traditional Chinese are semantically consistent.

## 1. Introduction

Chinese is usually written in two forms: simplified Chinese (mainly used in Mainland China and Singapore) and traditional Chinese (mainly used in Hong Kong, Macao, and Taiwan). Although simplified Chinese is derived from traditional Chinese, the two systems are quite different on various levels, such as character set, encoding method, orthography, vocabulary, and semantics, which create barriers to communication between different areas where Chinese is spoken. This linguistic phenomenon is due to the independent development of these two homologous systems in the past half century, and they will continue to evolve in their respective cultural environments. However, in the past few decades, with the increase in exchange activities between four cross-strait regions, the problem of conversion between simplified Chinese and traditional Chinese as well as the comparison of the differences between simplified Chinese and traditional Chinese has attracted the attention of more and more researchers [1–4]. In short, the comparison between Simplified Chinese and Traditional Chinese has important reference value for the study of language evolution.

So far, research on comparing differences between these two forms of Chinese still focuses on the character or lexical levels [1, 3, 5]. For example, Fei [6] made a systematic comparison of the similarities and differences of the current Chinese characters in simplified and traditional Chinese characters; Li [7] made an in-depth analysis of the reasons for the differences in the form of simplified and traditional Chinese characters from the aspects of politics, history and culture, and the principles of character selection; Liu [8] conducted a comprehensive analysis mainly from the perspective of eliminating the differences in form; Jiang [9] mainly compared and analyzed simplified and traditional Chinese vocabulary from two aspects: homographs with different meanings and different forms with synonymous meanings; Li and Qiu [10] discussed the causes, types, and processing methods of differences in dictionaries across the Taiwan Strait.

On the other hand, as an important methodology for linguistic research, complex networks-based approaches show their advantage in revealing the global features of language which have been successfully applied to analyse languages at various levels, e.g., lexical [11–13], word co-occurrence [14–18], syntax [19–21], and semantic [22–24]. This is because language is a typical hierarchical system which has a highly complex network structure, and complex network analysis methods have the advantage of revealing the laws of language as a whole. Hence, in this paper, we apply complex network analysis methods to explore the differences between simplified and traditional Chinese character systems from a holistic perspective. Specially, according to the construction method of the word co-occurrence network, this paper proposed to construct simplified Chinese and traditional Chinese word co-occurrence networks with different numbers of nodes and different corpus sizes and then make corresponding research on the complex characteristics of these networks. Through the obtained simplified and traditional Chinese core dictionary, we explored the differences between the two languages. In addition, this paper proposed to use primitives representing language semantics to analyze the semantic differences between simplified and traditional languages.

The rest of this paper is organized as follows.  Section 2 introduces the related work.  Section 3 puts forward a brief introduction to some basic concepts related to complex network analysis. Then, in Section 4, we constructed networks with different text scales and carried out corresponding studies on the characteristics of complex networks, e.g., cumulative degree distribution, clustering coefficient, kernel lexicon, and motif analysis. Finally, Section 5 concludes the paper.

## 2. Related Work

At present, the comparison and analysis of the differences between simplified and traditional Chinese mainly remain at the level of character shapes or words. The main reason why readers find it difficult to read unfamiliar written materials in simplified or traditional characters is due to the difference in glyphs. Studies have shown that the actual number of characters that can be compared in the simplified and traditional Chinese character lists is 4,786 [6]. Among them, 41% of the simplified and traditional characters used in mainland China and Taiwan have the same glyph, totaling 1,947 characters; 24% of the similar glyphs, totaling 1,170 characters; and 35% of different glyphs, totaling 1,669 characters. Simplified and traditional Chinese belong to the same ancestor and developed from the same ancient Chinese. Therefore, the differences between simplified and traditional Chinese need to be compared and analyzed systematically and comprehensively from the perspective of the language as a whole, which explores the differences between the two written forms of Chinese development status and law. However, the current comparative work of simplified and traditional Chinese characters has only achieved outstanding achievements on the level of character form and word, while other language levels (such as semantics and syntax) have not been involved.

As a typical hierarchical system, language exhibits a highly complex network structure at all levels (phonetics, morphology, syntax, and semantics) [25]. At present, a lot of research studies have been carried out on the complex characteristics of language networks on different levels, including lexical or vocabulary networks, word or character co-occurrence networks, and syntactic networks, the semantic networks. These research studies are important for identifying and understanding the topological structure of language. Among them, the research studies of Chinese network mainly include the following: in terms of morphology or vocabulary network, Li et al. [13] used Chinese characters as nodes based on the principle that two Chinese characters can form words and constructed a Chinese phrase network and studied the dynamic characteristics of the phrase network; in terms of syntactic network, Liu [20] used the syntactic labeling tree bank to connect the words with syntactic relations and finally established the Chinese syntactic dependency network and explored the complex network characteristics of the syntactic network; in the semantic network (current research studies on Chinese semantic networks are still relatively small), Liu et al. [24] constructed a small semantic network to explore the complex characteristics of the Chinese semantic network; and Cancho and Solé [14] used the English-speaking country corpus to construct an English word co-occurrence network and found that the English language network has a small world and scale-free features. Liu and Sun [15] used the same construction method to construct a simplified Chinese word co-occurrence network. The experiment proved that the simplified Chinese word co-occurrence network has complex network characteristics similar to the English word co-occurrence network. Other works [12, 26, 27] used different construction strategies to construct a Chinese word, word co-occurrence network, and English word co-occurrence network based on different themes of Chinese and English (prose, novels, popular science articles, and news reports) corpora.

## 3. Foundations

In this section, some basic concepts are put forward.  Section 3.1 describes the basic definitions of the complex network. Then, Section 3.2 describes small-world networks and scale-free networks. Finally, Section 3.3 gives a brief introduction of motif analysis.

### 3.1. Basic Definitions

In general, a network *G* can be denoted as a two-tuples *(V, E)*, where *V* is the set of vertices and *E* is the set of edges. In a language network, a vertex *v*_*i*_(1 ≤ *i* ≤ |*V*|) may represent a radical, character, or word; and an edge *e*_*ij*_(1 ≤ *i*, *j* ≤ |*V*|) can characterize the relationship between *v*_*i*_ and *v*_*j*_.

Given a network, the conventional indicators, such as average path length, clustering coefficient, degree distribution, and cumulative degree distribution, are used to specify its statistical characteristics. These indicators could be defined, respectively, as follows:

Average Path Length ($\bar{d}$): the average distance between two reachable vertices:

(1) $$\begin{array}{c} \bar{d}=\frac{2}{N\left(N-2\right)}\sum_{i>j}d_{ij}, \end{array}$$

where *N* is the number of vertices in the network, *d*_*ij*_ is the distance between vertex *v*_*i*_ and vertex *v*_*j*_ which also means the number of edges in the shortest path linking them.

Clustering Coefficient (*C*): the percentage of the neighbours that two vertices share. The clustering coefficient of vertices *i* could be defined as follows [23]:

(2) $$\begin{array}{c} C_{i}=\frac{2E_{i}}{k_{i}\left(k_{i}-1\right)},\;k_{i}\neq 0,1, \end{array}$$

where *k*_*i*_ is the degree of vertex *i* and *E*_*i*_ is the number of edges among the vertices in the nearest neighbourhood of vertex *i*. Moreover, the clustering coefficient of the whole network is the average of all individual *C*_*i*_, as follows:

(3) $$\begin{array}{c} C=\frac{1}{N}\sum_{i=1}C_{i}. \end{array}$$

### 3.2. Small-World Networks and Scare-Free Networks

A complex network is called a small-world network, in which the average number of edges lying between any two vertices is very small, while the clustering coefficient remains large. Specifically, for an ER random network in a small-world network, *d*_*ER*_ and *C*_*ER*_ represent the average shortest path and clustering coefficient, respectively, and *d* is similar to *d*_*ER*_, but *C* ≫ *C*_*ER*_ [28].

The degree distribution reveals the distribution of vertices by degree:

(4) $$\begin{array}{c} P\left(k\right)=\sum_{k^{\prime }=k}^{\infty }P\left(k^{\prime }\right), \end{array}$$

and the percentage of the vertices whose degrees are *k* is represented as *P (k)*:

(5) $$\begin{array}{c} P\left(k\right)=\sum_{k^{\prime }=k}^{\infty }{k^{\prime }}^{-\gamma }\propto k^{-\left(\gamma -1\right)}. \end{array}$$

Under certain circumstances, a network is called scale-free if it fits the power law well and lies between 2 and 3 [29].

### 3.3. Motif Analysis

Motif, a subgraph constructed by a few edges and vertices, was first used in biological academic area [30]. For a complex network, a motif represents a subnetwork containing a small number of nodes and edges. Biemann et al. [31] first applied motif analysis in linguistic networks and semantic features to explore the difference between natural language text and text generated by an *N*-gram language model in terms of semantic characteristics.

**Figure 1:**
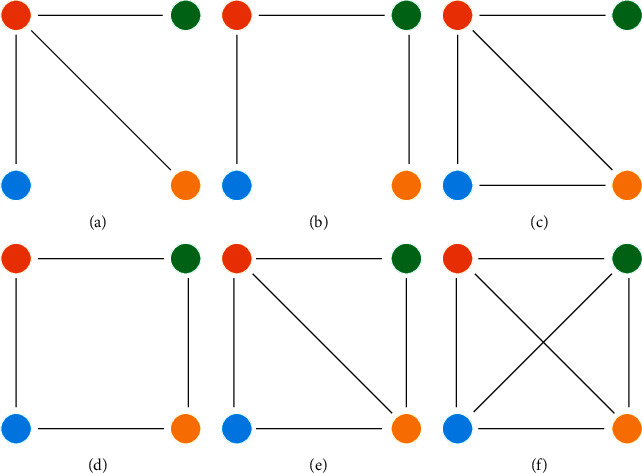
All undirected motifs of size 4. (a) Star; (b) chain; (c) 3-loop-out; (d) box; (e) semiclique; (f) Clique.

Besides, motif analysis involves an intermediate level of a network, which specifically means to count the motif constructed by *n* nodes to approach comparison among networks. As to undirected co-occurrence networks, *n* is usually at least 3. A 3-node motif is a triple-contained completely in calculating the clustering coefficient. Therefore, we use 4-node motif analysis to compare the semantic differences of co-occurrence networks. All six kinds of undirected 4-node motifs are shown in Figure 1.

## 4. Experimental Comparisons

This section addresses the experimental comparisons between simplified and traditional Chinese based on methods from complex network science.  Section 4.1 describes the dataset used as well as the construction of the word co-occurrence networks. Then, Sections 4.2–4.4 describe the comparisons on small-world and scale-free, kernel lexicons, and motif analysis, respectively.

### 4.1. Dataset and Network Construction

In this experiment, texts from *Chinese GigaWord Third Edition (LDC2007T38)*(https://catalog.ldc.upenn.edu/LDC2007T38) are used as the experimental materials, of which the simplified Chinese texts are from “*Xinhua News Agency*” (hereinafter referred to as XIN) and the traditional Chinese texts are from “*Central News Agency*” (hereinafter referred to as CNA).

Based on the datasets, word co-occurrence networks are built according to the method proposed by [32]. Concretely, words in the texts are regarded as nodes in the networks, and any two nodes are connected if the distance of the corresponding words is not greater than 2.

After the networks are constructed, their statistical properties are observed and compared. Please note that, only the networks built from the similar text scales are compared which avoids the influence of the text scales. In this experiment, three text scales are used, and the statistics of all the networks are shown in Table 1. For the co-occurrence network of simplified and traditional Chinese words under the same corpus scale, we designed three sets of experiments. The scales of the corpus used in these three sets increased from initial 7 million words to 10 million words and then 15 million words.

### 4.2. Small-World and Scare-Free

Given the built networks, we use a complex network analysis tool, *Pajek*^*2*^ to calculate the statistical properties of the networks.  Table 2 shows the results.

From Table 2, we can find that all the networks satisfy $\bar{d}\approx d_{ER}$ and C≫*C*_*ER*_, which means that all the networks are small-world networks. However, it could also be observed that the average degrees of traditional networks are about 5 points larger than those of the corresponding simplified networks. The possible reason is the many-to-one mappings between traditional Chinese and simplified Chinese, i.e., different words in |traditional Chinese have the same forms. For example, two traditional Chinese words “編制 (biān zhì)” and “編製 (biān zhì)” have that same form “编制 (biān zhì)” in simplified Chinese. It is the many-to-one mappings between traditional Chinese and simplified Chinese lead to larger numbers of nodes, edges, and average degrees.

**Figure 2:**
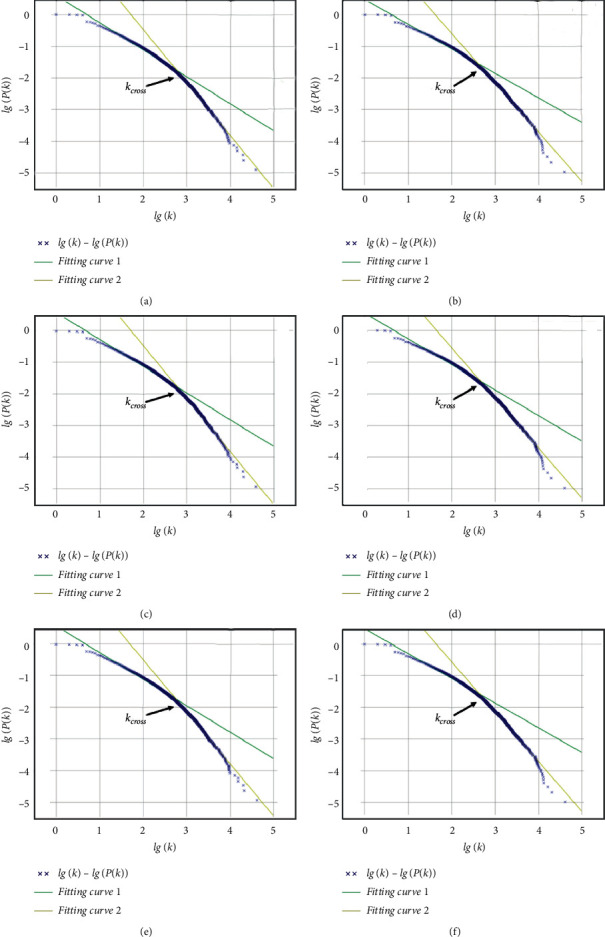
Cumulative degree distributions of all the built networks. (a) XIN_1_. (b) CNA_1_. (c) XIN_2_. (d) CNA_2_. (e) XIN_3_. (f) CNA_3_.

Moreover, we plot the cumulative degree distributions of all the networks, as well as their fitting curves in Figure 2. It is clear that both traditional and simplified Chinese networks fit the power law well. In addition, the power-law exponents of all the networks belong to the range of 2 and 3, indicating that all of the networks are scale-free.

### 4.3. Kernel Lexicons

By observing the cumulative degree distribution curves in Figure 2, we can learn that the scattered points can be fitted by two lines with different slopes. And the whole data set is divided into two parts at the crossover point. The more frequently a word is used in daily life, the more semantic meanings it may contain [33]. And the frequency *f* of a given word is relevant to its degree *k*, as follows:

(6) $$\begin{array}{c} k\propto f^{\alpha },\;\alpha >0. \end{array}$$

Followed [15], we may obtain a kernel dictionary by sorting words according to their degrees and selecting those with more degrees. Concretely, the capacity of kernel lexicons is calculated as follows:

(7) $$\begin{array}{c} N_{KL}=\;N\times P\left(k_{\text{cross}}\right), \end{array}$$

where *N* denotes the number of nodes, or specifically the number of words, and *k*_cross_ denotes the percentage of the words whose degrees are not less than *k*_cross_, which is the number at the crossover point.

Table 3 shows the sizes of the constructed kernel lexicons. From Table 3, we can learn that the sizes are all about 10^3^ levels and satisfy the claim proposed by [15, 34]. However, we observed the number of traditional Chinese kernel lexicons is much greater than that of simplified Chinese. Concretely, the traditional Chinese kernel lexicons are about 900 words, which are more than simplified Chinese in average.

To find out the possible reasons, we further analysis the part-of-speech tags and the lengths for the words in the kernel lexicons. The results are listed in Tables 4 and 5, respectively.

From Table 4, we found that, both forms of Chinese have a large proportion on entity words (noun and verb) whose orders are roughly the same. The percentage of verb in traditional Chinese is generally greater than that in simplified Chinese, indicating that verb weakening is an important development process in simplified Chinese.

From Table 5, we learned that kernel lexicons extracted from the traditional Chinese corpora contain more 1-character words than the ones extracted from the simplified Chinese corpora. This implies that traditional Chinese maintains some features of classical Chinese, while simplified Chinese does not.

### 4.4. Motif Verification

Followed [31], we performed the motif analysis upon each networks constructed in Section 4.1. The results are shown in Table 6. There is no difference between simplified Chinese networks and the corresponding traditional Chinese networks, except that the traditional Chinese complex networks tend to have more motifs than the simplified Chinese ones which is due to the larger number of nodes and edges of the traditional Chinese networks. This shows that simplified and traditional Chinese are consistent on the semantics level.

### 4.5. Example Comparison

We found that parts of speech of these different words are mainly reflected in nouns, verbs, time words, gerunds, adverbs, numerals, and ground nouns, as shown in Table 4. Among them, nouns, verbs, gerunds, and adverbs vary with corpus. However, there are also some words that are unique or frequently used in specific areas due to regional and political reasons, such as “总统”, “中华民国'”, “卫生署”, “社会主义”, and “农民工”; time words, numerals, and geographical nouns also have different usage habits or frequency of use due to different regional cultures, such as “二零零五年”, “2005年”, “二十五”, “25”, “高雄县”, and “长江”.

Furthermore, we found that nearly 25% of the different words in traditional Chinese are single-character words, such as “逾/vg”, “採/v”, “恆/ag”, and “常/d”. The number of single-character words in different words in simplified Chinese is relatively small. These single-character words frequently appear in the traditional corpus. Some words are function words or substantive words with grammatical effect, and some words are produced by the word segmentation tool incorrectly. But most single-character words appear in sentences mainly in the form of classical Chinese, “黃金/n 博物/n 園區/n 為/v 將/p 此/rz 深/d 具/vg 教育/vn 意義/n 的/uj 活動/vn 推廣/v 至/p 瑞芳/ns 在地/b 的/uj 學校/n 與/c 社區/n 團體/n ” and “他/rr 一度/d 懷疑/v 自己/rr 能否/v 常/d 保/v 早先/t 的/uj 成就/n”. This shows that many ancient Chinese words still appear in the written language of the traditional Chinese character system with a higher frequency, i.e., the written language of the traditional Chinese character system retains more classical Chinese characteristics.

In summary, the core dictionaries of the simplified and traditional Chinese character systems have a certain degree of versatility. However, in the process of language development, there have been some differences due to regional usage habits, environment, politics, and the generation of new words. In addition, in the development of the traditional Chinese character system, its written language still retains certain characteristics of classical Chinese.

## 5. Conclusion

In this paper, we proposed complex network to explore differences between simplified Chinese and traditional Chinese. To the best of our knowledge, this is the first work to use complex network-based approaches in comparing differences between simplified and traditional Chinese. Through the comparisons, we achieve 3 interesting results. Firstly, both co-occurrence networks for simplified and for traditional Chinese are small-world and scale-free networks. However, given the same corpus scale, the co-occurrence networks for traditional Chinese tend to have larger number of nodes, which may be due to the numerous one-to-many character/word mappings from simplified Chinese to traditional Chinese. Secondly, the kernel lexicons of traditional Chinese have more entries than those of simplified Chinese, which may be because that, in traditional Chinese, more ancient Chinese words are kept while less weak verbs are used. Thirdly, the motif analysis shows that there are no differences between the simplified Chinese networks and the corresponding traditional Chinese ones. In other words, simplified Chinese and traditional Chinese are semantically consistent.

## Figures and Tables

**Table 1:** Statistics of the built word co-occurrence networks. XIN_1_, XIN_2_, and XIN_3_ are from different parts of the XIN dataset; CNA_1_, CNA_2_, and CNA_3_ are from different parts of the CNA dataset.

	Theme (name)	Text scales (# of words) (M)	Sources	# of nodes
Group 1	XIN_1_	55.9	XIN (Jan., 2006–May., 2006)	1.06*∗*10^5^
	CNA_1_	55.3	CNA (Jan., 2006–Mar., 2006)	1.14*∗*10^5^
Group 2	XIN_2_	79.8	XIN (Jan., 2006–Jun., 2006)	1.26*∗*10^5^
	CNA_2_	79	CNA (Jan., 2006–Apr., 2006)	1.38*∗*10^5^
Group 3	XIN_3_	115	XIN (Jan., 2006–Sep., 2006)	1.52*∗*10^5^
	CNA_3_	114	CNA (Jan., 2006–May., 2006)	1.69*∗*10^5^

**Table 2:** Properties of the built networks. *N*: number of nodes; *E*: number of edges; $\bar{k}$: average degree of nodes; *C*: clustering coefficient; $\bar{d}$: average path length among reachable pairs of nodes; *C*_*ER*_: clustering coefficient of an *ER* network with same numbers of nodes and edges; *d*_*ER*_: average path length among reachable pairs of nodes in an ER network with same numbers of nodes and edges; and *γ*: power-law exponent in equation (5).

Metric	Dataset theme					
	XIN_1_	CNA_1_	XIN_2_	CNA_2_	XIN_3_	CNA_3_
*N*	1.06*∗*10^5^	1.14*∗*10^5^	1.26*∗*10^5^	1.38*∗*10^5^	1.52*∗*10^5^	1.69*∗*10^5^
*E*	0.27*∗*10^7^	0.32*∗*10^7^	0.35*∗*10^7^	0.41*∗*10^7^	0.45*∗*10^7^	0.53*∗*10^7^
$\bar{k}$	50.01	55.08	54.45	59.39	58.45	62.86
C	0.68	0.68	0.69	0.70	0.72	0.73
$\bar{d}$	2.69	2.72	2.69	2.73	2.70	2.74
*C*_*ER*_	4.69*∗*10^−4^	4.80*∗*10^−4^	4.28*∗*10^−4^	4.30*∗*10^−4^	3.90*∗*10^−4^	3.70*∗*10^−4^
*d*_*ER*_	3.24	3.21	3.26	3.20	3.25	3.20
*γ*	2.17	2.18	2.16	2.17	2.15	2.15

**Table 3:** Word length statistics in kernel lexicons (%).

	*k*_cross_	*P* (*k*_cross_)	NKL
XIN_1_	606	0.01470	1,193
CNA_1_	420	0.02399	2,205
XIN_2_	622	0.01442	1,187
CNA_2_	494	0.02073	1,944
XIN_3_	581	0.01613	1,350
CNA_3_	466	0.02207	2,121

**Table 4:** Comparison on part-of-speech statistics (%).

Metric	Dataset theme					
	XIN_1_	CNA_1_	XIN_2_	CNA_2_	XIN_3_	CNA_3_
Noun	28.83	31.25	29.06	31.07	27.85	31.40
Verb	23.22	26.94	22.91	27.11	22.52	27.11
Adverb	6.87	3.53	6.74	7.00	7.11	6.51
Numeral	4.78	3.36	4.80	3.34	4.67	3.25
Gerund	4.44	3.67	4.38	4.38	3.78	3.30
Time	5.11	3.40	5.22	2.52	5.04	2.69
Noun of Place	3.69	3.31	3.88	2.88	4.96	3.30
Adjective	2.68	2.77	2.78	2.62	2.62	2.83
Quantifier	3.10	2.49	2.95	2.62	2.96	2.50
Preposition	3.35	2.22	3.29	2.52	3.04	2.36
Conjunction	2.01	2.04	2.02	2.16	2.07	2.07
Noun of Locality	2.18	1.72	2.19	1.90	2.30	1.74

**Table 5:** Word length statistics in kernel lexicons (%).

Word length	Dataset theme					
	XIN_1_	CNA_1_	XIN_2_	CNA_2_	XIN_3_	CNA_3_
1	25.40	27.76	24.26	28.24	24.96	28.52
2	68.73	66.85	69.17	67.28	67.41	66.20
3	5.11	4.85	5.73	4.12	6.15	4.86
4	0.34	0.27	0.34	0.21	0.89	0.19
5	0.42	0.27	0.51	0.15	0.59	0.24

**Table 6:** Comparison on motif analysis (%).

Word length	Dataset theme					
	XIN_1_	CNA_1_	XIN_2_	CNA_2_	XIN_3_	CNA_3_
Star	93.7959	91.3591	93.7661	91.2177	93.7512	91.1679
Chain	3.4099	4.8152	3.3738	4.7887	3.3632	4.7131
TLO	2.5563	3.4790	2.6098	3.6182	2.6256	3.7186
Box	0.0328	0.0493	0.0332	0.0512	0.0335	0.0523
SCQ	0.1875	0.2725	0.1980	0.2959	0.2059	0.3162
Clique	0.0172	0.0246	0.0188	0.0281	0.0202	0.0316

TLO: three-loop-out. SCQ: semiclique.

## Data Availability

The data used can be accessed at https://catalog.ldc.upenn.edu/LDC2007T38.

## References

[1] Wang L., Wang X., Wu J. (2013). The correspondence simplified characters and traditional characters and the mutual conversion. *Journal of Chinese Information Processing*.

[2] Zhenjun P., Tianfang Y. (2015). Chinese characters conversion system based on lookup table and statistical methods. *Computer Engineering and Applications*.

[3] Dai H. (2016). Linguistic analysis of the intelligent conversion system of simplified and traditional Chinese characters text. *Liaoning Normal University (Social Science Edition)*.

[4] Wang L. (2020). Review of and reflections on the hot topics in the application of contemporary Chinese charactersl Chinese characters text. *Applied Linguistics*.

[5] Li M.-H., Wu S.-H., Zeng Yi-C., Yang P.-C., Ku T. (2010). Chinese characters conversion system based on lookup table and language model. *Computational Linguistics and Chinese Language Processing*.

[6] Fei J. (1993). Comparative analysis of current Chinese characters across the Taiwan straits. *Language Application*.

[7] Li L. (1998). An analysis of the reasons for the differences in the forms of Chinese characters on both sides of the Taiwan straits. *Journal of Guangxi University*.

[8] Liu X. (2007). Study on the unification of Chinese characters across the Taiwan straits.

[9] Jiang Y. (2006). Differences in Chinese vocabulary between the two sides of the taiwan straits and their reasons. *Jimei University Journal*.

[10] Li X., Qiu Z. (2012). Definement and treatment of difference words in cross-strait dictionaries-new problems in cross-strait co-edited Chinese dictionaries. *Language Application*.

[11] Motter A. E., De Moura A. P. S., Lai Y.-C., Dasgupta P. (2002). Topology of the conceptual network of language. *Physical Review E*.

[12] Li Y., Wei L., Li W., Niu Y., Luo S. (2005). Small-world patterns in Chinese phrase networks. *Chinese Science Bulletin*.

[13] Li J., Zhou J., Luo X., Yang Z. (2012). Chinese lexical networks: the structure, function and formation. *Physica A: Statistical Mechanics and Its Applications*.

[14] Cancho R. F. I., Solé R. V. (2001). The small world of human language. *Proceedings of the Royal Society of London. Series B: Biological Sciences*.

[15] Liu Z.-Yuan, Sun M.-Song (2007). Chinese word cooccurrence network: its small world effect and scale-free property. *Journal of Chinese Information Processing*.

[16] Zhou S., Hu G., Zhang Z., Guan J. (2008). An empirical study of Chinese language networks. *Physica A: Statistical Mechanics and Its Applications*.

[17] Liang W., Shi Y., Tse C. K., Liu J., Wang Y., Cui X. (2009). Comparison of co-occurrence networks of the Chinese and English languages. *Physica A: Statistical Mechanics and Its Applications*.

[18] Liu H., Li W. (2010). Language clusters based on linguistic complex networks. *Chinese Science Bulletin*.

[19] Cancho R. F. I., Solé R. V., Köhler R. (2004). Patterns in syntactic dependency networks. *Physical Review E*.

[20] Liu H. (2008). The complexity of Chinese syntactic dependency networks. *Physica A: Statistical Mechanics and Its Applications*.

[21] Liu Z.-Y., Zheng Y.-b., Sun M.-S. (2008). Complex network properties of Chinese syntactic dependency network. *Complex Systems and Complexity Science*.

[22] Steyvers M., Tenenbaum J. B. (2005). The large-scale structure of semantic networks: statistical analyses and a model of semantic growth. *Cognitive Science*.

[23] Wang X. F., Xiang Li, Chen G. R. (2006). *Theory of Complex Networks and its Application*.

[24] Liu H. (2009). Statistical properties of Chinese semantic networks. *Science Bulletin*.

[25] Solé R. V., Corominas-Murtra B., Valverde S., Steels L. (2010). Language networks: their structure, function, and evolution. *Complexity*.

[26] Sigman M., Cecchi G. A. (2002). Global organization of the wordnet lexicon. *Proceedings of the National Academy of Sciences*.

[27] Li Y., Wei L., Niu Y., Yin J. (2005). Structural organization and scale-free properties in Chinese phrase networks. *Chinese Science Bulletin*.

[28] Watts D. J., Strogatz S. H. (1998). Collective dynamics of “small-world”networks. *Nature*.

[29] Barabási A.-L., Albert R. (1999). Emergence of scaling in random networks. *Science*.

[30] Shen-Orr S. S., Milo R., Mangan S., Alon U. (2002). Network motifs in the transcriptional regulation network of escherichia coli. *Nature Genetics*.

[31] Biemann C., Roos S., Weihe K. (2012). Quantifying semantics using complex network analysis. *Proceedings of Coling 2012*.

[32] Cancho R. F. I., Solé R. V. (2001). Two regimes in the frequency of words and the origins of complex lexicons: zipf’s law revisited. *Journal of Quantitative Linguistics*.

[33] Griffin Z. M., Bock K. (1998). Constraint, word frequency, and the relationship between lexical processing levels in spoken word production. *Journal of Memory and Language*.

[34] Dorogovtsev S. N., Mendes J. F. F. (2001). Language as an evolving word web. *Proceedings of the Royal Society of London. Series B: Biological Sciences*.

